# Supplementary material for: Machine learning-enabled forward prediction and inverse design of 4D-printed active plates
Source: Nat Commun. 2024 Jun 29;15:5509. doi: 10.1038/s41467-024-49775-z (PMC11217466; doi:10.1038/s41467-024-49775-z)
Supplement: Supplementary file 1 — Supplementary Information [file 41467_2024_49775_MOESM1_ESM.pdf]

Supplementary Information for

Machine learning-enabled forward prediction and inverse design of 4D-printed active plates

Xiaohao Sun[1$], Liang Yue[1$], Luxia Yu[1$], Connor T. Forte[1], Connor D. Armstrong[1], Kun Zhou[2], Frédéric Demoly[3,4], Ruike Renee Zhao[5], H. Jerry Qi[1]*

[1]The George W. Woodruff School of Mechanical Engineering, Georgia Institute of Technology, Atlanta, GA 30332, USA

[2]Singapore Centre for 3D Printing, School of Mechanical and Aerospace Engineering, Nanyang Technological University, 50 Nanyang Avenue, Singapore 639798, Singapore

[3]ICB UMR CNRS 6303, Belfort-Montbeliard, UTBM, 90010 Belfort, France

[4]Institut universitaire de France (IUF)

[4]Department of Mechanical Engineering, Stanford University, Stanford, CA 94305, USA

[$]These authors are equal contribution first authors.

*Corresponding author: qih@me.gatech.edu

Supplementary Note 1. Additional boundary conditions evaluated

In addition to the original and converted BCs, we study the other two BCs that allow for the free shape morphing of plates, referred to as BC3 and BC4, to gain more insights into how BCs affect the ML model performance, thus aiding in selecting the optimal BC settings. The BC3 is described by the following,

$$\begin{aligned} & x_A = y_A = z_A = 0, \\ & y_B = z_B = 0, \\ & z_D = 0. \end{aligned} \tag{1}$$

The BC4 is described by the following,

$$\begin{aligned} & x_A = y_A = z_A = 0, \\ & y_B = z_B = 0, \\ & z_C = 0. \end{aligned} \tag{2}$$

The ML model performance on these two BCs is shown in **Figure 3c**. The two additional BCs show very similar performance to the original BCs for either deep (ResNet-33) or shallow (ResNet-7) model. The converted BCs, which mimic a *corner-clamped* condition, show significantly improved performance in the ResNet-33 model. This improvement is attributed to the resulting spatially sequential dependency in plate deflection.

Supplementary Note 2. Dataset preparation and statistics

Example patterns for the fully random and island datasets are shown in **Supplementary Figure 1a**. The generation of the fully random dataset is simply through the function *randint* of NumPy library, without any pattern constraints. The generation of island datasets involves two steps. First, random binary island images are generated by growing on random seeds. **Supplementary Figure 1b** shows an example growth process with three seeds (5 × 5 image used for clarity). In each growth step, one of neighbors (light green) of existing seed or island phases (dark green) is randomly picked to join the island phase. One island image is generated once the island phase(s) reaches a certain fraction, which is randomly picked between 0.1 and 0.52. Second, the island images are self-combined or inter-combined to form the material distribution designs. Specifically, an image *I* and its negation not-*I* can be combined with each other or with all "0" and "1" images, giving 5 designs. Two randomly sampled images, *I* and *J*, can be combined with their negations, not-*I* and not-*J*, forming 4 designs. These combination operations are repeated to generate 25,000 island designs, and we obtain 56,250 random designs in total.

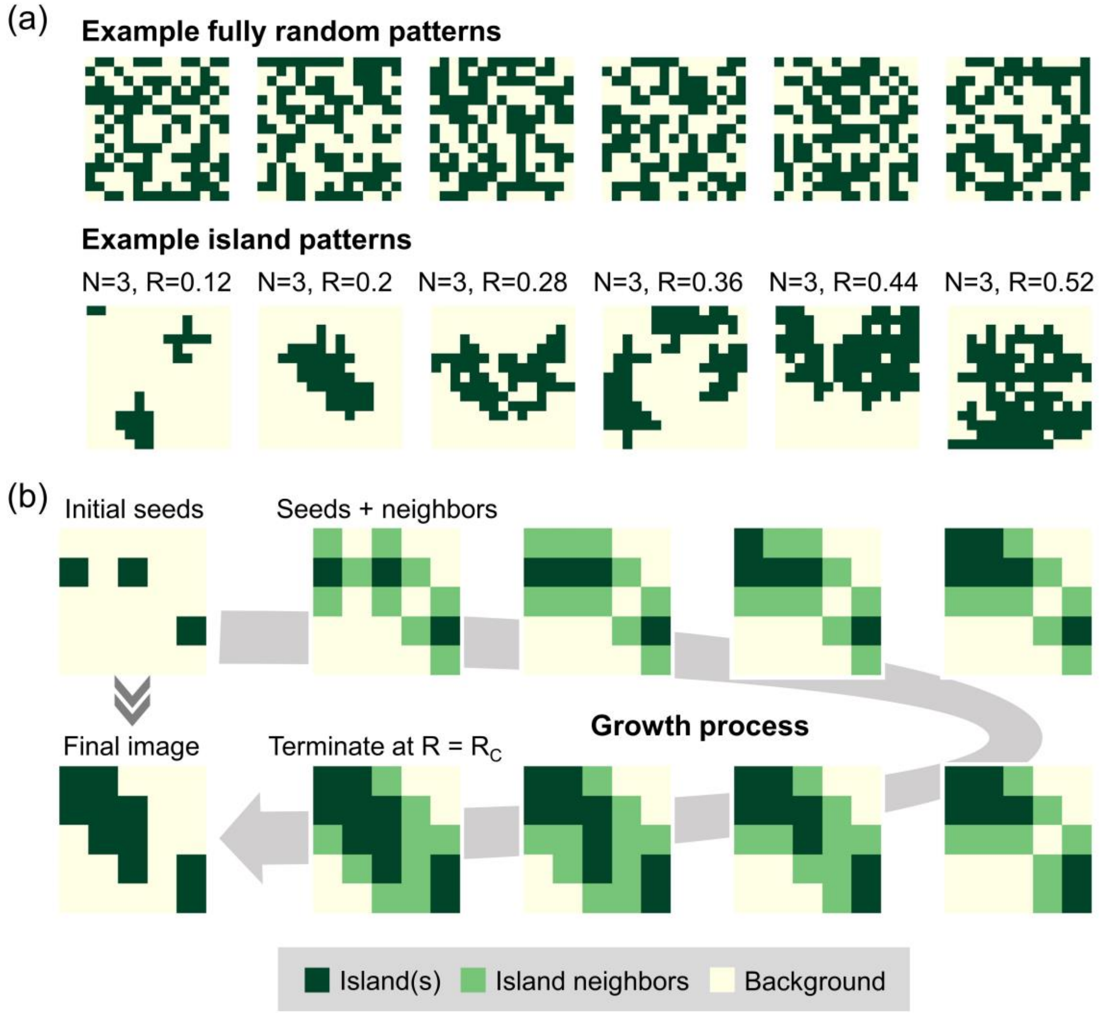

Supplementary Figure 1. (a) Example fully random patterns (top) and island patterns with random seeds and different island fractions (bottom). The "N" represents the number of initial seeds, and the "R" represents the island fractions. In this work, as a design has 15 × 15 × 2 voxels, images shown here are example patterns. (b) Example growth process for generating the island image. The 5 × 5 image is used for clarity. In each growth step, one of neighbors (light green) of existing seed or island phases (dark green) is randomly picked to join the island phase, and the growth terminates once the island fraction (R) reaches a certain value (R_C) and an island image is generated. To generate our actually used random island designs (15 × 15 × 2), we first create random island images by growing on random seeds and the R_C is randomly picked between 0.1 and 0.52. These island images are then self-combined or inter-combined to form the final designs, as described in Materials and Methods of the main text.

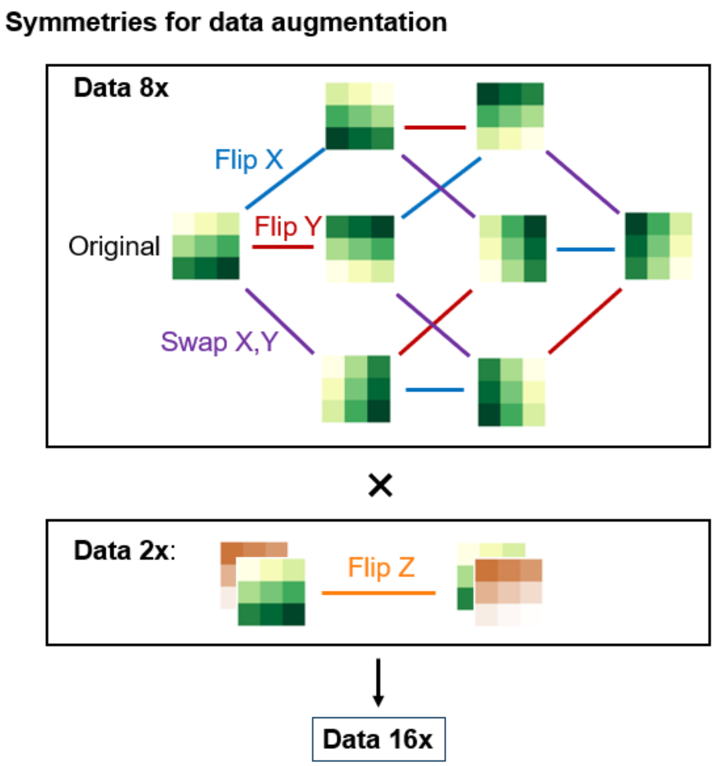

Supplementary Figure 2. Schematics of the symmetries and augmented designs. The original design is first augmented 8 times through the combination of the “Flip X”, “Flip Y”, and “Swap X,Y” operations, then augmented 2 times through the “Flip Z” operation. Upon each operation, the true actuated shape can be easily transformed from the shape of the design before the operation based on the original BCs (see Eq.(1) of the main text). More specifically, for “Flip X”, we reverse the order of all coordinates in the *x*-dimension, translate the shape (all coordinates) to the origin (0,0,0), mirror the coordinate *x* about the plane *x*=0, and rotate the shape about the *z*-axis to meet Eq.(1) to obtain the transformed shape. For “Flip Y”, we follow a similar procedure but use *y*-related operations taking place of *x*-related ones. For “Swap X,Y”, we transpose the *x*- and *y*- dimensions of all coordinates and swap coordinates data of *x* and *y*. For “Flip Z”, we simply mirror the coordinate *z* about the plane *z*=0. Therefore, using the FE simulated shapes of the original design, the shapes of all augmented designs can be calculated accordingly. Eventually, the 16 times augmented dataset are obtained.

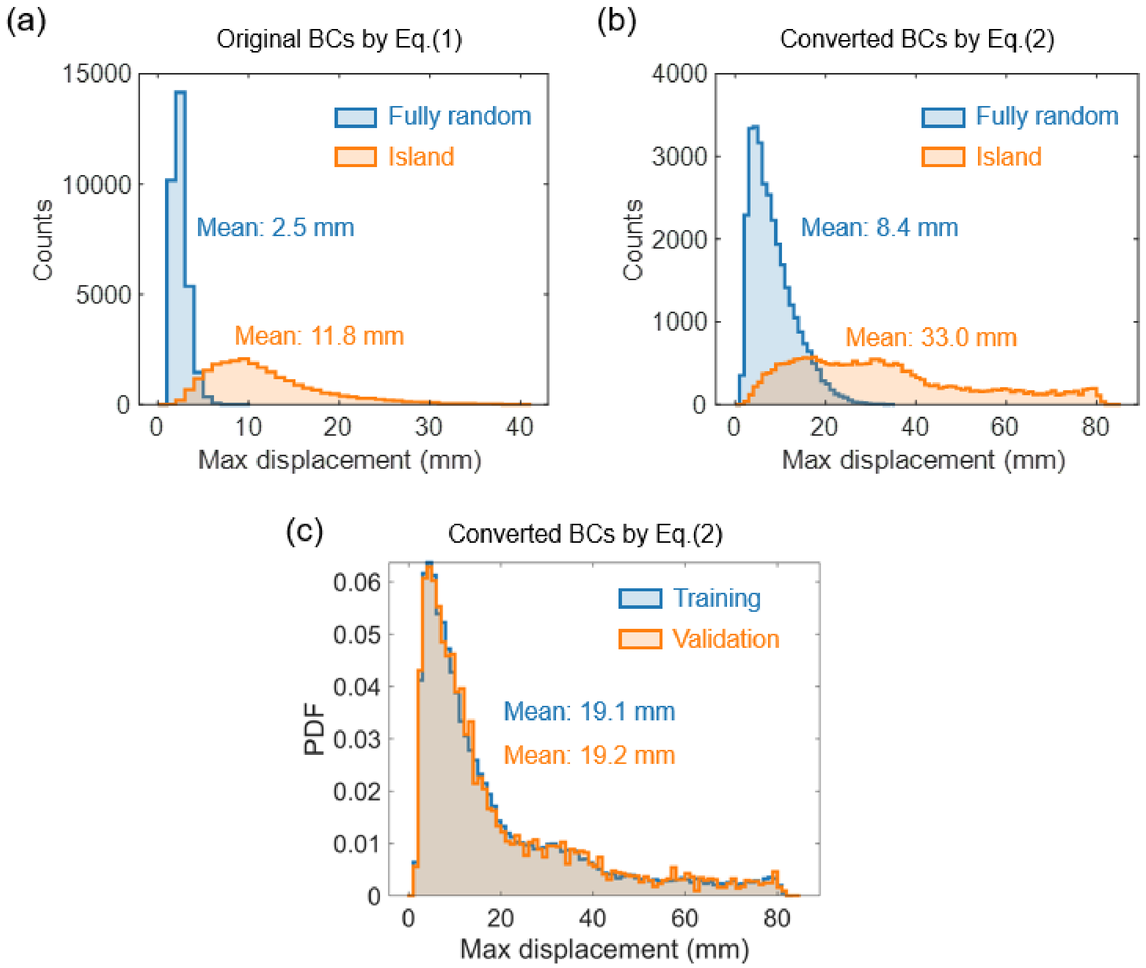

Supplementary Figure 3. The statistics of the dataset is studied. We calculate the maximum displacement of each data (design-shape pair) of the entire dataset and then compare the distributions of the maximum displacement (a-b) between the fully random dataset and the island dataset under (a) original BCs and (b) converted BCs, and (c) that between the training and validation datasets under converted BCs. In (a-b), the comparison is made on the data before augmentation; the count distribution is shown to visualize the fractions of two types of datasets (indicated by the area of the shaded region). In both sets of BCs, the island dataset overall exhibits a larger maximum displacement than that of the fully random dataset. In converted BCs, for example, the mean value of the maximum displacements is 8.4 mm for the fully random dataset and 33.0 mm for the island dataset, respectively. This implies large displacements compared to the edge length (40 mm). In (c), the probability density function (PDF) is shown to facilitate the comparison as the sizes of the two datasets are different (9:1). The result shows that the training and validation datasets follow similar distributions, which is expected since the two datasets are randomly sampled from the entire dataset.

Supplementary Note 3. Effects of bottleneck designs on ResNet performance

Supplementary Figure 4 shows the performance in terms of validation loss for ResNet with or without bottleneck designs, which are often used in deep ResNet. The result shows that the bottleneck design does not improve the model performance for the studied problem.

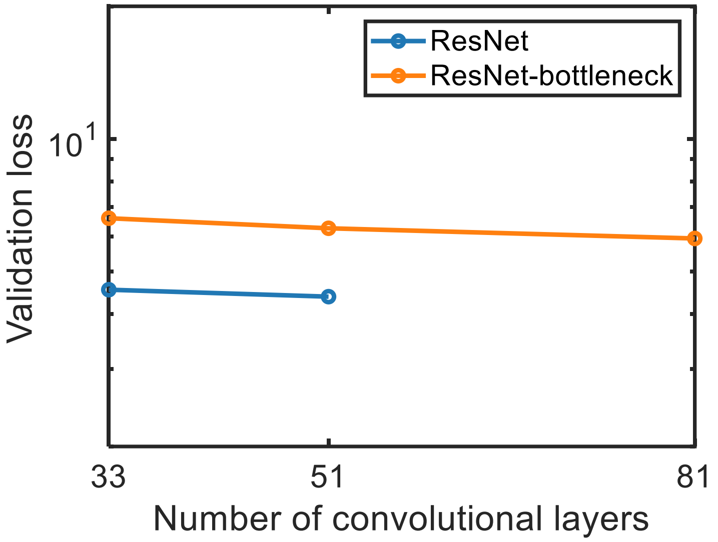

Supplementary Figure 4. Performance of ResNet with or without bottleneck designs versus the number of convolutional layers.

Supplementary Note 4. Graph convolutional network (GCN) performance

In addition to plain CNN and ResNet, we study the graph convolutional networks (GCNs) for our shape-change prediction tasks. We consider GCN models with different depths and architectures based on references [1-3]. **Supplementary Figure 5a** shows the schematic of a GCN architecture, which is comprised of multiple graph convolutional layers (GCLs) and skip connections (SCs). In GCN, the SC has a similar function to that in ResNet. Regarding the hyperparameter, we study the GCNs with different numbers of GCLs (or depth), hidden sizes, and numbers of SCs. In addition, we study the GCN with initial residual and identity mapping (GCNII) [3], which can be seen as a GCN with specially designed SCs. All GCN models are implemented in MATLAB, which are modified from (https://www.mathworks.com/help/deeplearning/ug/node-classification-using-graph-convolutional-network.html).

In our study, GCNs incorporating at least one SC consistently outperforms those without SCs. Thus, we only present the results of GCNs with SCs here, as shown in **Supplementary Figure 5b**. The GCN-21×64 (depth × hidden size) with 4 SCs is the top performer among the tested GCNs. However, its validation MSE is higher (indicating lower accuracy) than that of the best-performing CNN and ResNet models (see **Figure 3d**). Further, the regression plots for the best-performing GCN and CNN models are shown in **Supplementary Figure 5c** and **5d**, respectively, revealing that GCNs indeed exhibit inferior performance compared to CNNs, and consequently, to ResNets as well (see **Figure 3h**). The higher performance of CNN and ResNet are attributed to the structured nature of our voxel-level input and output data which aligns well with the convolutional filter’s capabilities. When dealing with non-structured data, such as structures with irregular geometries or topologies, GCNs might be appropriate. This will be explored in our future studies.

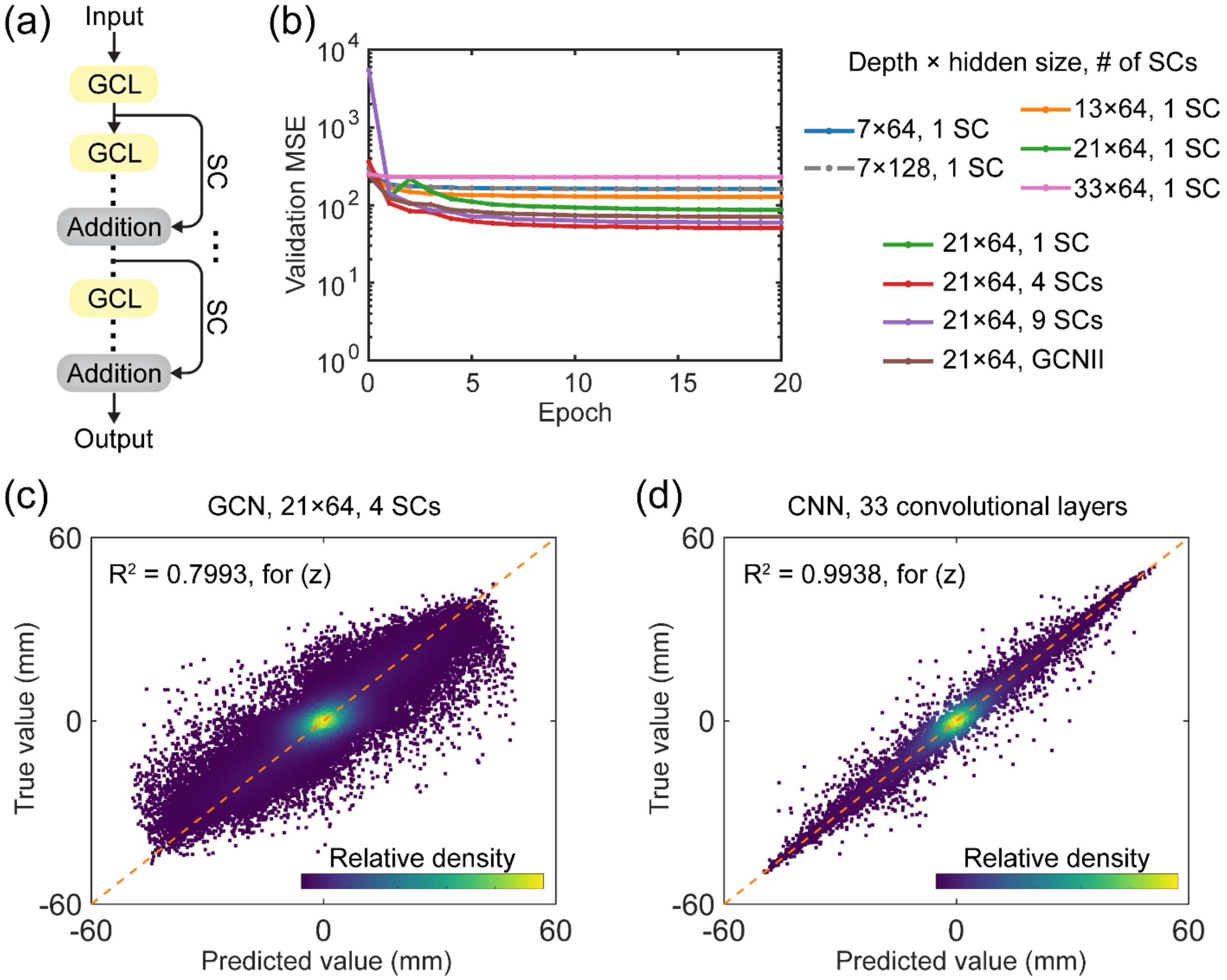

Supplementary Figure 5. Effects of model architectures. (a) Schematics of GCN architectures. GCL: graph convolutional layer. SC: skip connection. (b) Validation loss versus epochs showing the training progress of GCN with different depths, hidden sizes, and number of skip connections (SCs). GCNII is an extension of GCN with initial residual and identity mapping [3]. (c-d) Density scatter plots of the true versus ML-predicted coordinate *z* using the (c) GCN and (d) CNN with optimal hyperparameters.

Supplementary Note 5. Additional types of datasets

We expand our dataset beyond the original fully random and island patterns to include additional datasets with new pattern types. First, we introduce a hierarchical pattern type inspired by Buehler and colleagues[4]. These hierarchical patterns utilize "super" voxels composed of $i \times j$ ($i, j \geq 2$) true voxels, creating an $m \times n$ hierarchical grid for generating random designs. To cover a broad range of patterns, we develop a dataset with mixed hierarchies (named "HrchMix") by randomly sampling design hierarchies (m and n) from uniform distributions ($m, n \sim U(3, 7)$), followed by the generation of random grids ($\sum i = \sum j = 15$) random combination of random grids, and finally random assignment of two materials. The two layers of each material distribution can have different hierarchies to ensure diversity. In addition, we generate two independent validation datasets with the 3×3 and 5×5 hierarchies, where each "super" voxel contains 5×5 and 3×3 true voxels, respectively. Representative patterns for the HrchMix, 3×3, and 5×5 datasets are illustrated in **Supplementary Figure 6**. We also study their statistics and the results are shown in **Supplementary Figure 7**.

Second, we explore a completely different pattern type inspired by Spinodal decomposition[5], a process related to phase separation that results in distinctive patterns useful for material design. To cover a broad range of patterns, the spinodoid dataset (named "Spnd") was generated with varying anisotropies, orientations, and periods, using an approach modified from that described in [5]. The difference is that we generate random fields by sampling wave vectors from a unit circle in 2D, instead of a unit sphere in 3D as done in [5]. This helps to speed up the sampling process while also allowing for controls of the anisotropy, orientations and periods. The random fields are then binarized to obtain spinodoid patterns. The threshold is calculated using the density sampled from a uniform distribution $\sim U(0.45, 0.55)$. Finally, the spinodoid patterns are randomly self-

combined or inter-combined, using the same approach for the island dataset, to form the material distribution designs. Representative patterns for the Spnd datasets are illustrated in **Supplementary Figure 6**. We also study their statistics and the results are shown in **Supplementary Figure 7**.

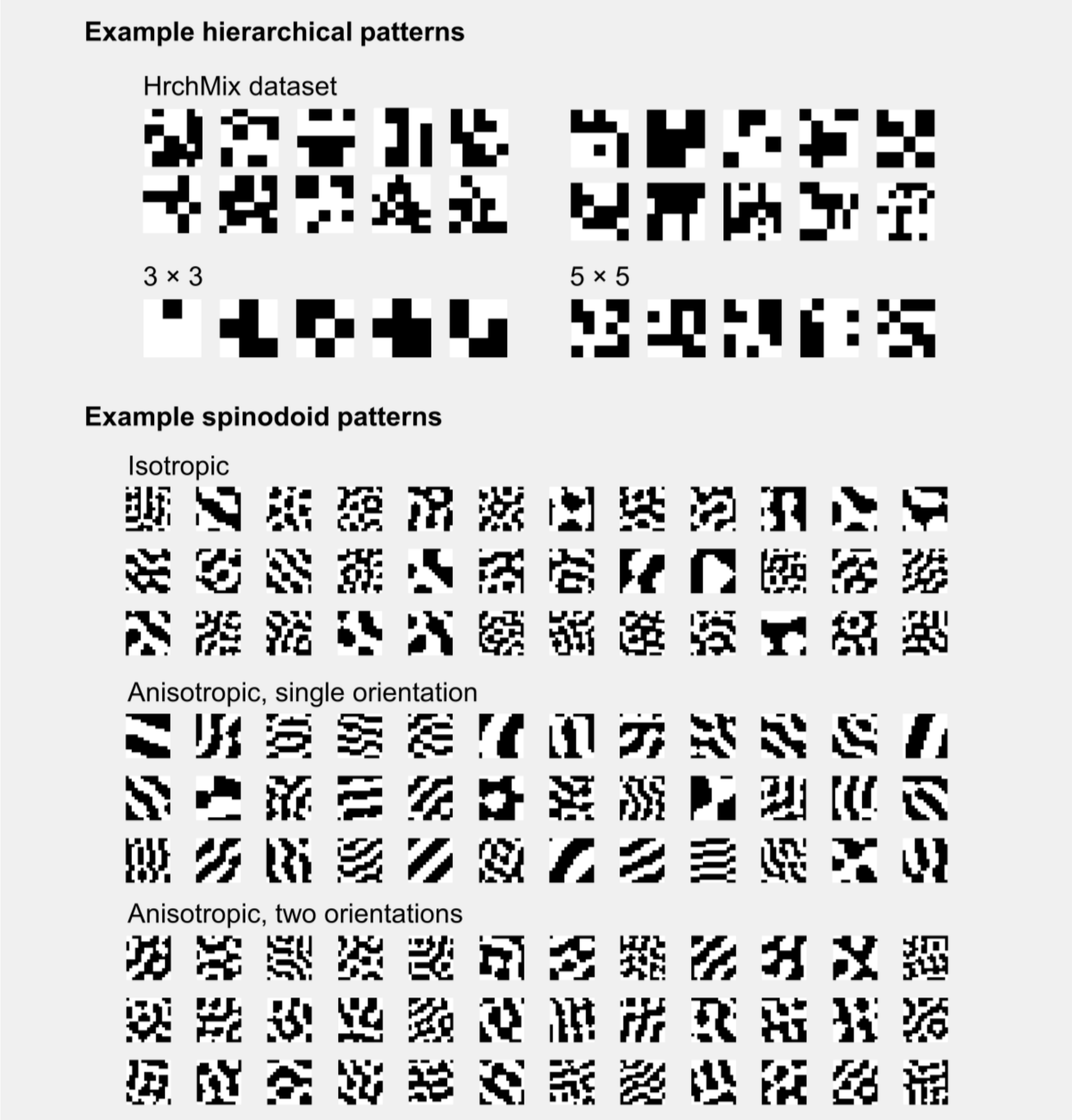

Supplementary Figure 6. Representative patterns for the hierarchical patterns and spinodoid patterns.

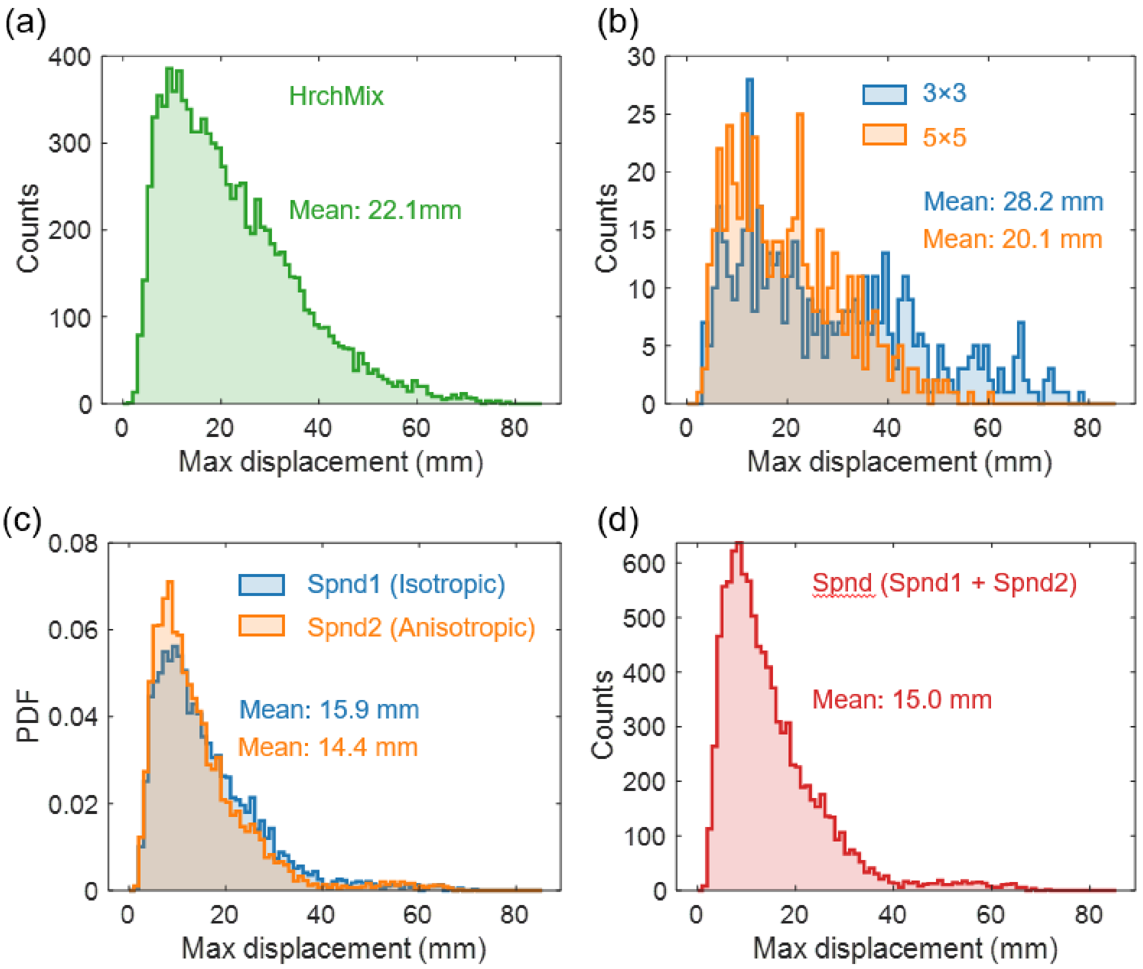

Supplementary Figure 7. The statistics of the newly generated datasets. We also calculate the maximum displacement of each datapoint (design-shape pair) of the entire dataset and then depict the distributions of the maximum displacement for (a) HrchMix dataset, (b) 3×3 and 5×5 datasets, (c) Spnd1 dataset generated with isotropic spinodoid patterns and Spnd2 dataset generated with anisotropic spinodoid patterns, and (d) Spnd dataset dataset. All datasets are based on the converted BCs. The mean value of the maximum displacements of each dataset is shown in the corresponding panel. These new datasets overall exhibit a maximum displacement between that of the fully random dataset and that of the island dataset.

The representative patterns and statistics of these new datasets confirm that they differ from the existing fully random and island datasets. We split the HrchMix and Spnd datasets into training and validation sets and incorporate the new training sets into our original training set, maintaining the overall size by replacing an equivalent number of

original datapoints. The combined training set is then used to train a new ResNet-based ML model, the performance of which, compared to the existing model across various validation sets (original, HrchMix, Spnd, 3×3 and 5×5), is shown in **Supplementary Figure 8**. First, our existing ML model shows an excellent performance on the new datasets, demonstrating its strong ability to generalize. Second, incorporating these new dataset types into the training set improves the model's performance on the independent 3×3 and 5×5 validation sets, indicating an enhancement in generalization ability. Due to the slight improvement of the new model and the excellent performance of the existing model, we opt not to rerun the inverse designs with the new model.

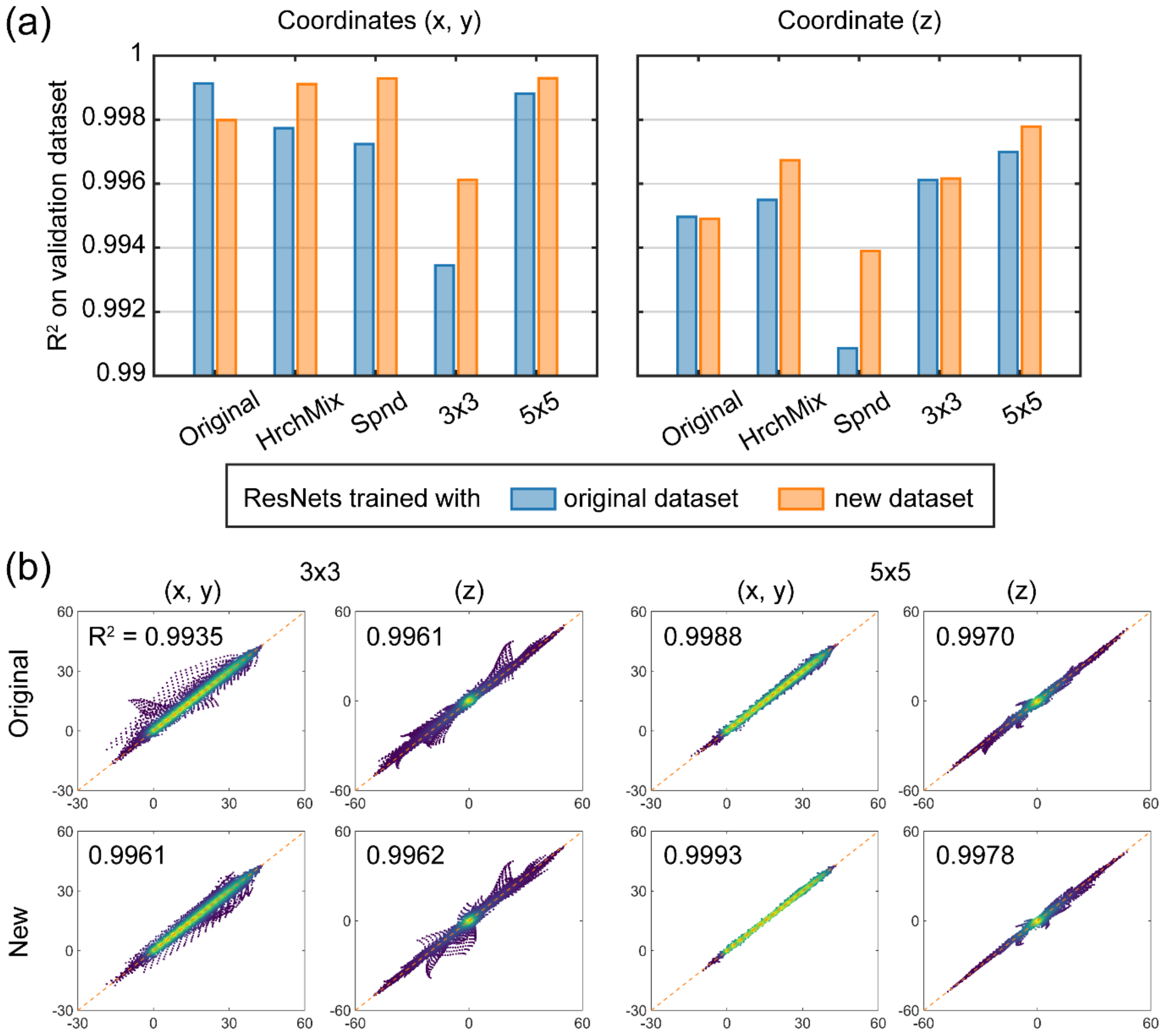

Supplementary Figure 8. Effects of new dataset types on the ML model's performance. (a) Performance of two ML models on different validation sets in terms of the R^2 value. (b) Performance of two ML models on two independent validation sets.

Supplementary Note 7. Methods for the ML-EA design

The EA procedure is schematically illustrated in **Figure 4b**. The EA starts by creating a population of random designs (or called individuals) in terms of digitally encoded arrays of '1's and '0's. The digital encodings are called genes. The EA then iteratively evaluates and evolves the population over successive generations, in which good individuals survive and reproduce whilst bad individuals are eliminated, until the number of generations reaches N_{gen} or an acceptable solution is found. Section 4.6 describes the ML-based evaluation procedure. After evaluation, the EA creates an offspring population for the next generation based on individuals of the current population. Three types of 'children' are created: elite (5%), crossover (76%), and mutation (19%) children. The top 5% fittest individuals of the current population are elite, which directly survive to the next generation. To create the other two types of children, the 'parents', a group of better performing individuals are selected via the binary tournament technique, which contribute their genes to the offspring: crossover children are created by combining genes of pairs of parents, while mutation children are created by randomly changing the genes of single parents. Note that these EA operations can naturally enforce the constraint that a gene only takes the value of 0 or 1. This is an integer-value EA process. Our ML-EA optimizations are performed using Matlab.

Supplementary Note 8. Additional inverse design results

We study the performance of our design approach in more detail in this section. We consider three FE-derived targets that are shown in **Figure 4c** but present more complete design results here. **Supplementary Figure 9** shows the results for the first target (row 1 of **Figure 4c**), where we perform two separate global-subdomain trials, one with ML-GD for the global design and ML-EA for the subdomain design (**Supplementary Figure 9b**), the other with ML-EA for both design steps (**Supplementary Figure 9c**). Both two design trials achieve the shapes that agree well with the target, although the one with ML-GD followed by ML-EA is slightly better than the other. In either global ML-GD or global ML-EA, the subdomain optimization further improves the result. The identified subdomain with relatively large approximation errors is mainly concentrated in the free boundary regions (e.g., free corners) that are distant to the fixed boundary (bottom-left corner). This is beneficial for the design improvement in the subdomain step, as the spatially sequential dependency allows for local adjustments in the free boundary regions (or subdomain) without affecting shapes closer to the fixed corner. This phenomenon is also seen in, for example, the second target (row 2 of **Figure 4c**), as shown in **Supplementary Figure 10**, where the identified subdomain after global optimization is mainly near the free corners or free edges.

Although ML-GD is slightly better than ML-EA in the case of **Supplementary Figure 9**, this is not always true as detailed below. The performances of the two algorithms are compared for the third target (row 3 of **Figure 4c**) as shown in **Supplementary Figure 11**. As ML-GD may be affected by the initial solution, to make fair comparisons, four different initial solutions are used for ML-GD, i.e., all-passive (“0”s), all-active (“1”s), all-neutral (“0.5”s, which are physically meaningless), and random designs. The results show that the all five design trials, one ML-EA and four ML-GD trials, achieve the

shapes that agree remarkably well with the target (**Supplementary Figure 11**). However, the ML-EA achieves the best result for this target. In addition, the ML-GD optimal designs are indeed sensitive to the initial solution, which is expected for such a highly nonlinear inverse problem. The results for the two targets (**Supplementary Figure 9** and **11**) show that the performance of ML-EA and ML-GD is case-dependent, although both can achieve shapes that agree well with the target.

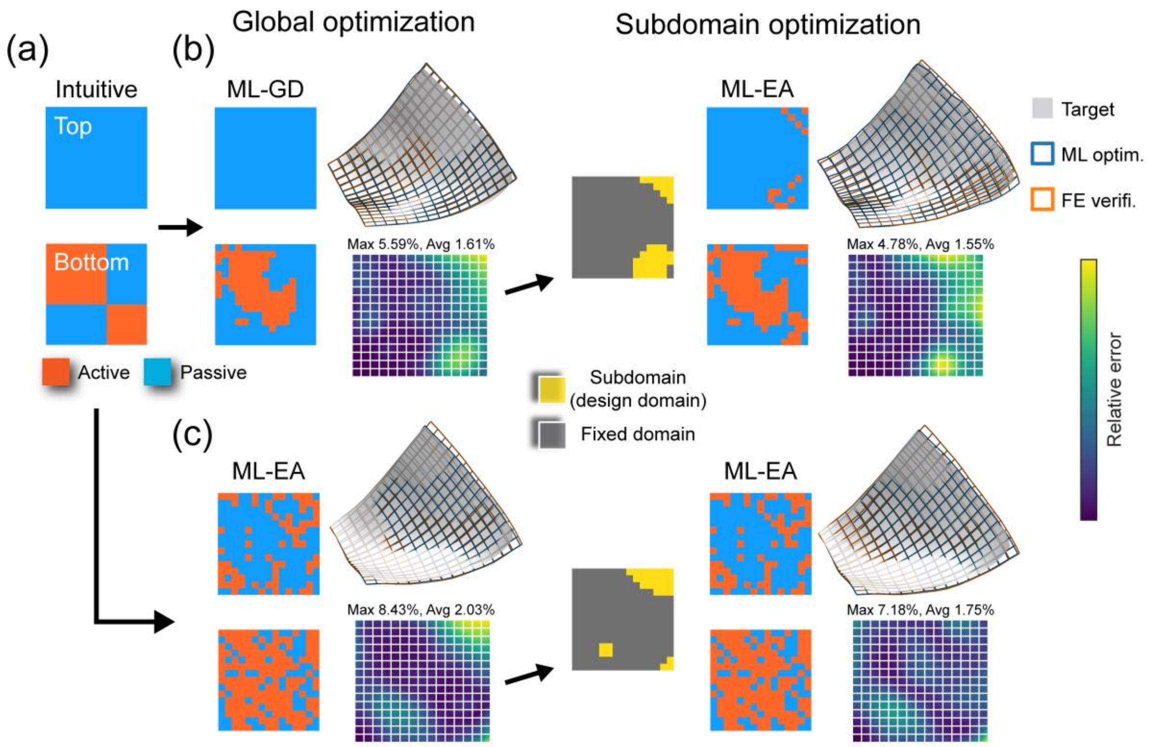

Supplementary Figure 9. Inverse design results for the FE-derived target shape based on given voxel patterns. (a) Intuitive pattern whose deformed shape is the target. (b) Design results of the global ML-GD followed by subdomain ML-EA. (c) Design results of the global ML-EA followed by subdomain ML-EA. The approximation error maps of the achieved shape (by FE) against the target relative to the edge length (40 mm) are shown.

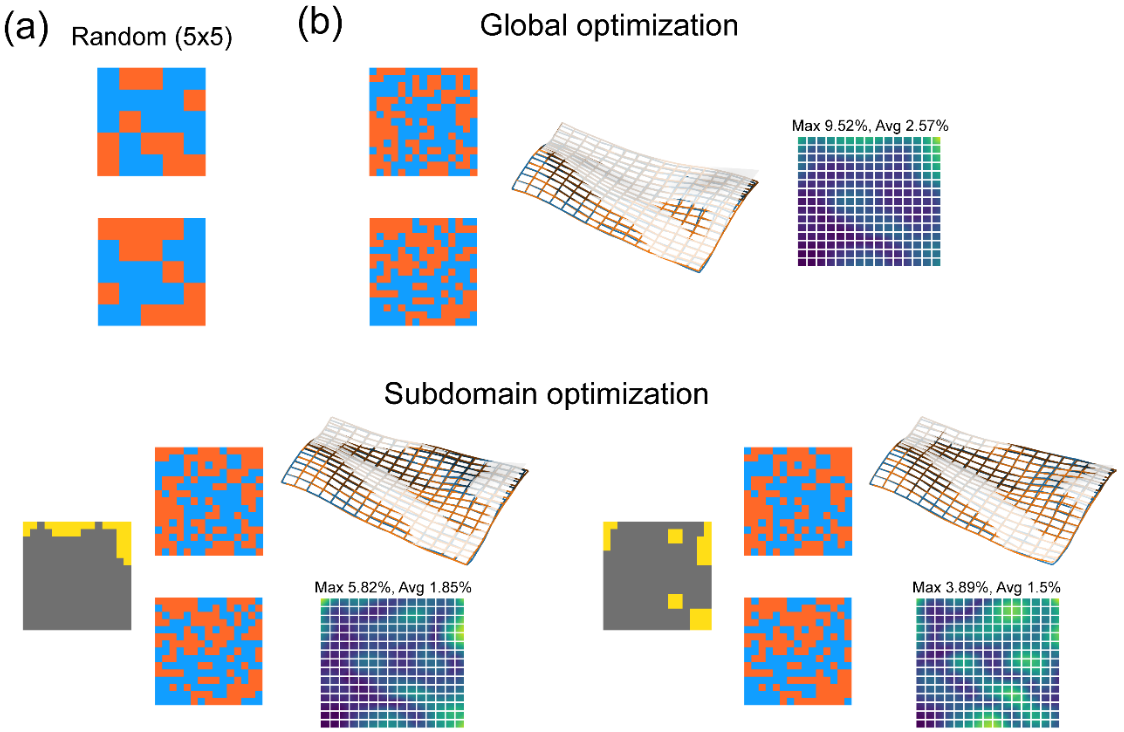

Supplementary Figure 10. Inverse design results for the FE-derived target shape based on given voxel patterns. (a) Random pattern whose deformed shape is the target. (b) Design results of the global ML-EA followed by two steps of subdomain ML-EA. The approximation error maps of the achieved shape (by FE) against the target relative to the edge length (40 mm) are shown.

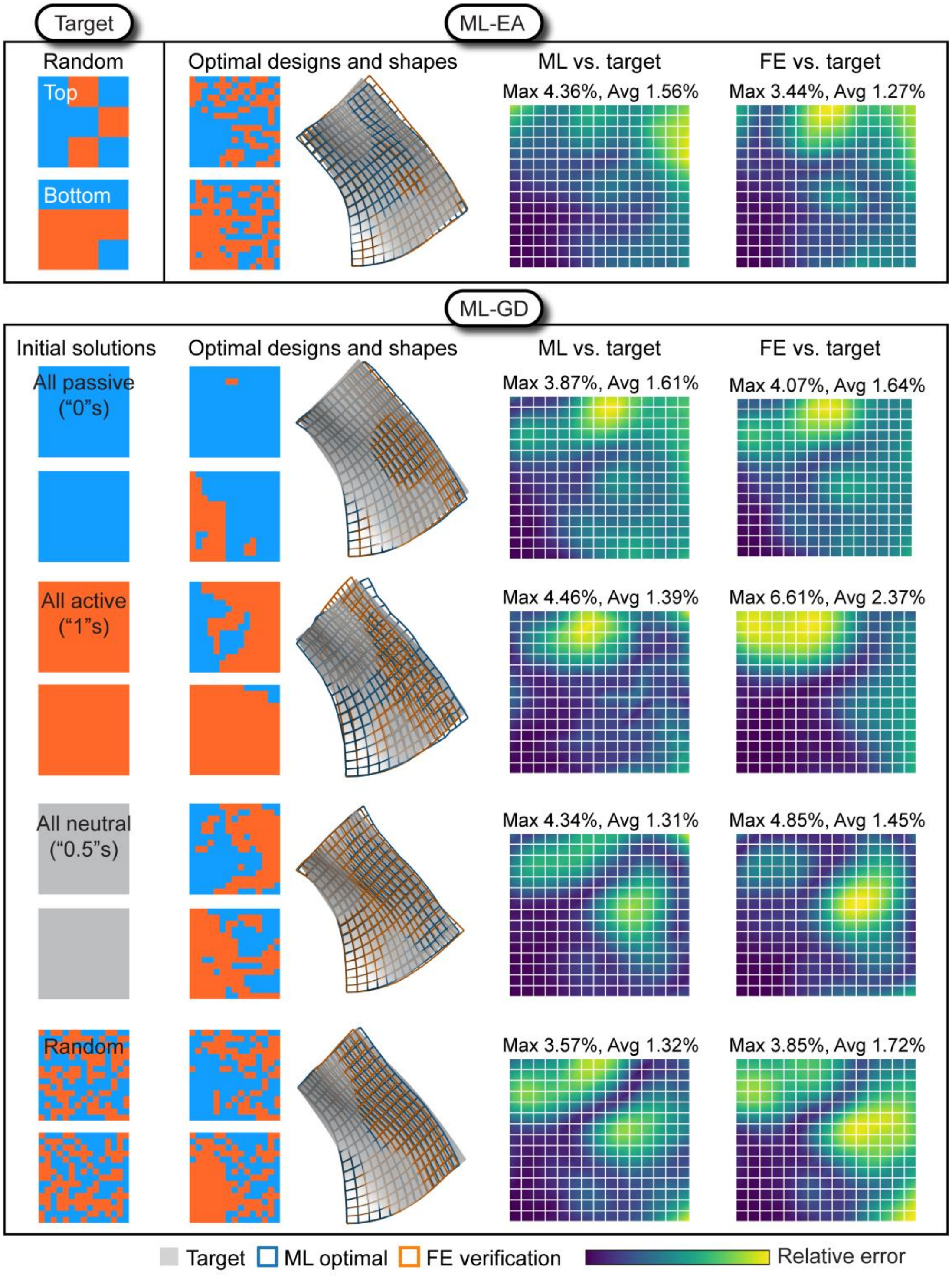

Supplementary Figure 11. Inverse design results for the FE-derived target shape based on given voxel patterns. We focus on the global optimization step and compare design results of the ML-EA and ML-GD. To make fair comparisons, four different initial solutions are used for ML-GD: all-passive ("0"s), all-active ("1"s), all-neutral ("0.5"s,

which are physically meaningless), and random designs. The approximation error maps of the achieved shapes (by ML and FE) against the target relative to the edge length (40 mm) are shown.

Supplementary Note 9. Tuning the print dimension to compensate for effects of property differences between the FE model and experiments

Mechanical properties in experiments are different from those used in the FE model for data generation. Specifically, the active ("1") and passive ("0") voxels are printed using brighter (0% grayscale, G0) and dimmer (60% grayscale, G60) lights and thus lead to higher-DoC and lower-DoC material phases, respectively. Experimental characterizations show that after volatilization, the G0 shows a modulus of 49.6 MPa and a shrinkage ratio of -0.12%, and the G60 material shows a modulus of 2.63 MPa and a shrinkage ratio of 5.54%. Thus, upon actuation, the two phases show a modulus ratio of 0.053 and a strain mismatch of 0.057. These two parameters are different from those of our FE model which uses an identical modulus (ratio=1) and a strain mismatch of 0.05.

Such a property difference between practical materials and the FE model will result in the difference in shape change between the ML prediction and 4D-printed part. This issue can be resolved by retraining a new ML model based on practical material parameters and rerunning the design, which, however, would require additional computational time and the new model is constrained to this material. Here, we use a strategy similar to that of our previous work (Ref. 40 of main text), i.e., tuning the print dimension to approximately compensate for this effect through an analytical model for the local curvature, without need for retraining.

We first briefly summarize the analytical curvature for a multi-layer, bi-phase beam with an eigenstrain mismatch as derived in our previous work (Section S7, Supporting Information of Ref. 40 of main text). As shown in **Supplementary Figure 12a**, the composite beam has N layers, each having a thickness of t/N. Two material phases are assigned to these layers. Let the modulus of the active and passive phase be E_1 and E_2 and

the volumetric strain be $\Delta\varepsilon$ and 0, respectively. Denoting the modulus and volumetric strain of layer i by $E^{(i)}$ and $\Delta\varepsilon^{(i)}$, respectively, and assuming the beam is unshearable, the longitudinal strain (x-direction) for an arbitrary point (position z) in layer i can be given by

$$\varepsilon^{(i)} = \varepsilon_0 - \Delta\varepsilon^{(i)} + \varepsilon_{bending}, \text{ with } \varepsilon_{bending} = -\frac{z - z_{NA}}{\rho}, \tag{3}$$

where the superscript (i) denotes a variable for layer i; ε_0 is the axial strain of the neutral axis (NA); $\varepsilon_{\text{bending}}$ is the bending strain; z_{NA} is the location of NA; ρ is the radius of curvature of NA with positive ρ meaning curling up. The equilibrium requires

$$\begin{aligned} &\sum_i \int_{layer(i)} E^{(i)} \varepsilon^{(i)} dz = 0 \\ &\sum_i \int_{layer(i)} E^{(i)} \varepsilon^{(i)} \left(z - z_{NA}\right) dz = 0 \end{aligned}. \tag{4}$$

Inserting (3) into (4) yields the curvature κ

$$\kappa = \frac{1}{\rho} = \frac{\sum_i E^{(i)} \left(-\Delta\varepsilon^{(i)} + \varepsilon_0\right)\left(\bar{z}^{(i)} - z_{NA}\right) t^{(i)}}{\sum_i E^{(i)} \left(\frac{1}{12}\left(t^{(i)}\right)^3 + t^{(i)} \left(\bar{z}^{(i)} - z_{NA}\right)^2\right)}, \tag{5}$$

with relevant variables given by

$$\begin{aligned} &\varepsilon_0 = \frac{\sum_i n^{(i)} \Delta\varepsilon^{(i)} t^{(i)}}{\sum_i n^{(i)} t^{(i)}} \\ &z_{NA} = \frac{\sum_i n^{(i)} \bar{y}^{(i)} t^{(i)}}{\sum_i n^{(i)} t^{(i)}} \\ &\bar{z}^{(i)} = t^{(1)} + \ldots + t^{(i-1)} + t^{(i)} / 2 \end{aligned} \tag{6}$$

where $n^{(i)} = E^{(i)}/E_1$; $t^{(i)} = (1+\Delta\varepsilon^{(i)})t/N$ is the thickness of layer i after expansion.

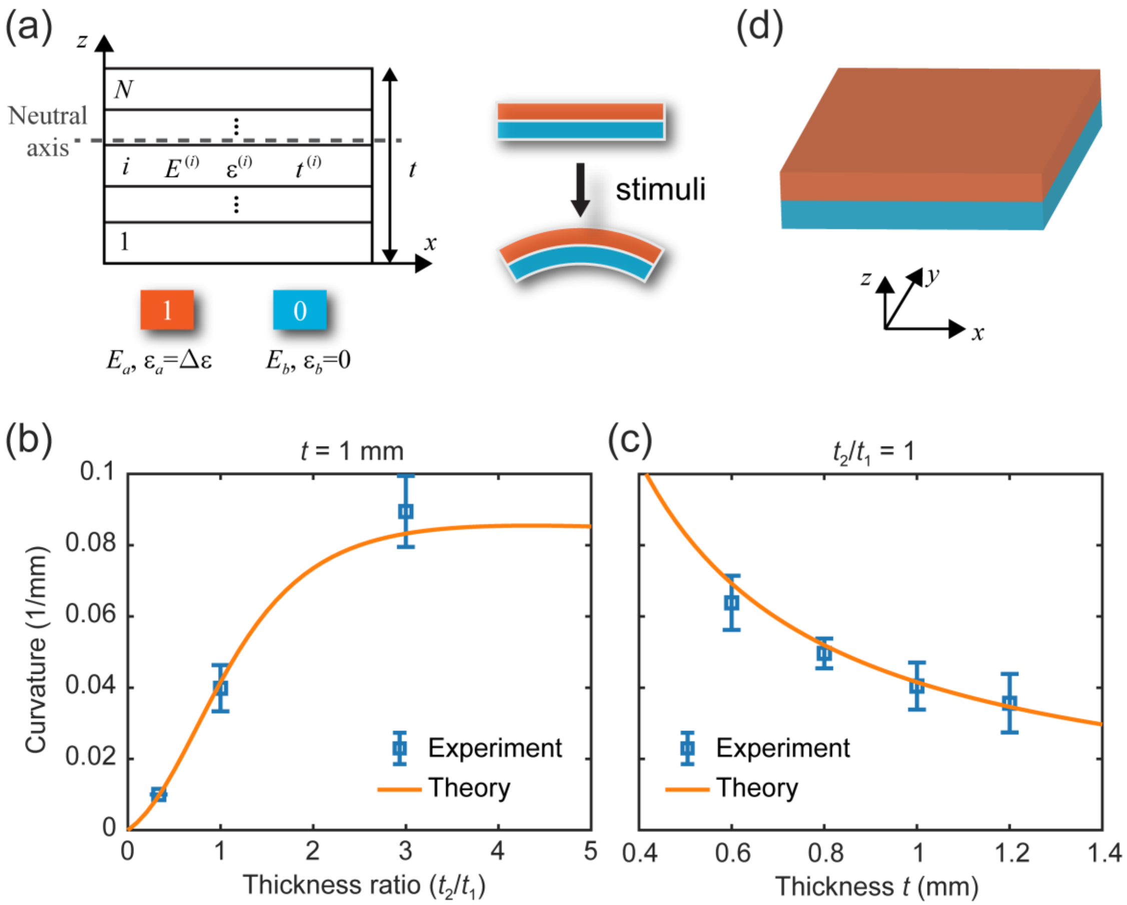

Supplementary Figure 12. (a) Schematic of a multi-layer bi-phase beam with a strain mismatch (left). Bending of a bilayer beam induced by the strain mismatch (right). (b) Comparison of theory and experiment for the relation between curvature and thickness ratio. (c) Comparison of theory and experiment for the relation between curvature and total thickness. (d) Schematic of a bilayer plate with a strain mismatch.

Next, we limit the multi-layer beam to a bilayer case and neglect the effect of strain mismatch on layer thickness, and then the curvature Eq.(4) becomes

$$\kappa = \frac{\Delta\varepsilon}{t}\bar{\kappa}(m,n), \text{ with } \bar{\kappa}(m,n) = \frac{6(1+m)^2}{3(1+m)^2 + (1+mn)\left(m^2 + \frac{1}{mn}\right)} \tag{7}$$

where $m=t_2/t_1$ is the thickness ratio, $n=E_2/E_1$ is the modulus ratio, $\bar{\kappa}(m,n)$ is the dimensionless curvature. As mentioned above, the experimentally printed materials

exhibit n_{Exp}=0.053 and $\Delta\varepsilon_{Exp}$ =0.057. Using these two values, we depict the analytical curvature versus thickness ratio and that versus total thickness in **Supplementary Figure 12b** and **12c**, respectively. To verify the analytical predictions, we print (1) beams with the identical thickness of 1 mm but having different thickness ratios and (2) beams with equal thickness for the two layers but having different total thicknesses, and measure their curvatures upon the actuation. In both cases, the experimental curvatures (symbols) are well predicted by the Eq.(7) (line), thereby confirming the validity of the analytical model (**Supplementary Figure 12b** and **12c**).

Now we return to the property difference between the FE model and printed materials and use the analytical model to calculate the resulting curvature difference between them. The FE model parameters (assumed in ML predictions) include m_{ML}=1, n_{ML}=1, $\Delta\varepsilon_{ML}$ =0.05, t_{ML}=1 mm, with which the curvature of a bilayer beam is calculated to be 0.075 mm^{-1}. For printed materials, using n_{Exp}=0.053 and $\Delta\varepsilon_{Exp}$ =0.057 and the same values for m and t, a lower curvature of 0.0416 mm^{-1} would be obtained. Note that the practical shape change depends on κL which scales linearly with $\Delta\varepsilon\bar{\kappa}\left(m,n\right)\frac{L}{t}$, where L can be taken as the edge length (L_{ML}=40 mm). We can thus tune the print dimension, i.e., the m_{Exp}, t_{Exp}, or the edge length L_{Exp}, such that

$$\kappa_{Exp}L_{Exp} = \kappa_{ML}L_{ML} \tag{8}$$

which is a condition for the printed part to show a similar shape with the ML prediction. In our 4D printing experiments, we adopt m_{Exp}=m_{ML}=1, t_{Exp}=0.6 mm, and L_{Exp}=42 mm to satisfy Eq.(8), as well as the manufacturability condition that each dimension of the design voxel (2.8 mm × 2.8 mm × 0.3 mm) are divisible by (0.05 mm)^3, the smallest voxel size in our DLP printing system.

It should be noted that the above analysis is for a composite beam. However, it is valid for bilayer plates (**Supplementary Figure 12b**) in the regime where the plate is under small-deflection, equi-biaxial bending without any instabilities. In this case, using σ_z=0, the Hooke's law becomes

$$\sigma_x = \frac{E}{1-\nu^2}\left(\varepsilon_x + \nu\varepsilon_y\right), \sigma_y = \frac{E}{1-\nu^2}\left(\varepsilon_y + \nu\varepsilon_x\right), \tag{9}$$

which, under an equi-biaxial condition, becomes decoupled,

$$\sigma_x = \sigma_y = \frac{E}{1-\nu}\varepsilon_x = \frac{E}{1-\nu}\varepsilon_y . \tag{10}$$

Using Eq. (10) and the equilibrium equations in two directions, a similar form as Eq.(7) can be derived, where the curvature κ represents two equal principal curvatures and n becomes $n = \left[E_2/(1-\nu_2)\right]/\left[E_1/(1-\nu_1)\right]$. The above assumption (small-deflection, equi-biaxial bending, no instabilities) is reasonable for a single design voxel (2.8 mm × 2.8 mm × 0.3 mm in experiments) under a relatively small, isotropic strain mismatch (0.057). When the plate involves a large island domain with distinct materials in two layers, the assumption may be invalid as the buckling may occur. A more accurate model will be explored in future studies. In the present work, our experimental results show that this dimension modification approach can approximately compensate for effects of the property difference between the FE model and experiments, offering an efficient way to expanding the applicability of ML model across different material systems and length scales.

Supplementary Note 10. Methods for algorithmically generated target surfaces

The surface equations x, y, and z are constructed from 2D parametric variables u and v that range between 0 and 1. For simplicity, all the complexity of the surface is confined to z, as such,

$$x(u,v) = u$$

$$y(u,v) = v$$

The equation for z is constructed from a group of simple functions with pre-defined boundary conditions and an additional polynomial for variation,

$$z_{bc}(u,v) = (u+v)(2-u-v)$$

$$h_{bc}(u,v) = \frac{u}{\sum_m a_m} + \frac{v}{\sum_n b_n}$$

$$P(u,v) = \left(\sum_{m=0}^{M} a_m u^m\right)\left(\sum_{n=0}^{N} b_n v^n\right)$$

$$= (a_0 + a_1 u + \cdots + a_M u^M)(b_0 + b_1 v + \cdots + b_N v^N)$$

$$z(u,v) = h \cdot z_{bc}(u,v) \cdot h_{bc}(u,v) \cdot P(u,v)$$

where h is a scaling factor. The zero-boundary condition, z_{bc} is such that,

$$z(0,0) = z(1,1) = 0$$

The height-boundary condition, h_{bc} allows,

$$z(1,0) = hb_0, \qquad z(0,1) = ha_0$$

Using the approach described above, we construct different target surfaces as given in **Table 3** of the main text.

Supplementary Note 11. Pre-smoothing of target shapes with unsmoothness features

We use a simple strategy to pre-smooth the target shape. Recall that an actuated shape is represented by the coordinates (x_{ij}, y_{ij}, z_{ij}) on all sampling points. The discrete Laplacian of, e.g., z, seen as a matrix, can be expressed as the following finite difference form,

$$\Delta z = z_{i-1,j} + z_{i+1,j} + z_{i,j-1} + z_{i,j+1} - 4z_{i,j} \quad (11)$$

Then the shape can be smoothed by iteratively performing the $z - \gamma\Delta z \rightarrow z$, where γ is an empirical factor. For the example shown in **Supplementary Figure 13**, γ is taken to be 0.01 and the numbers of iterations are 500 (bottom left) and 2000 (bottom right).

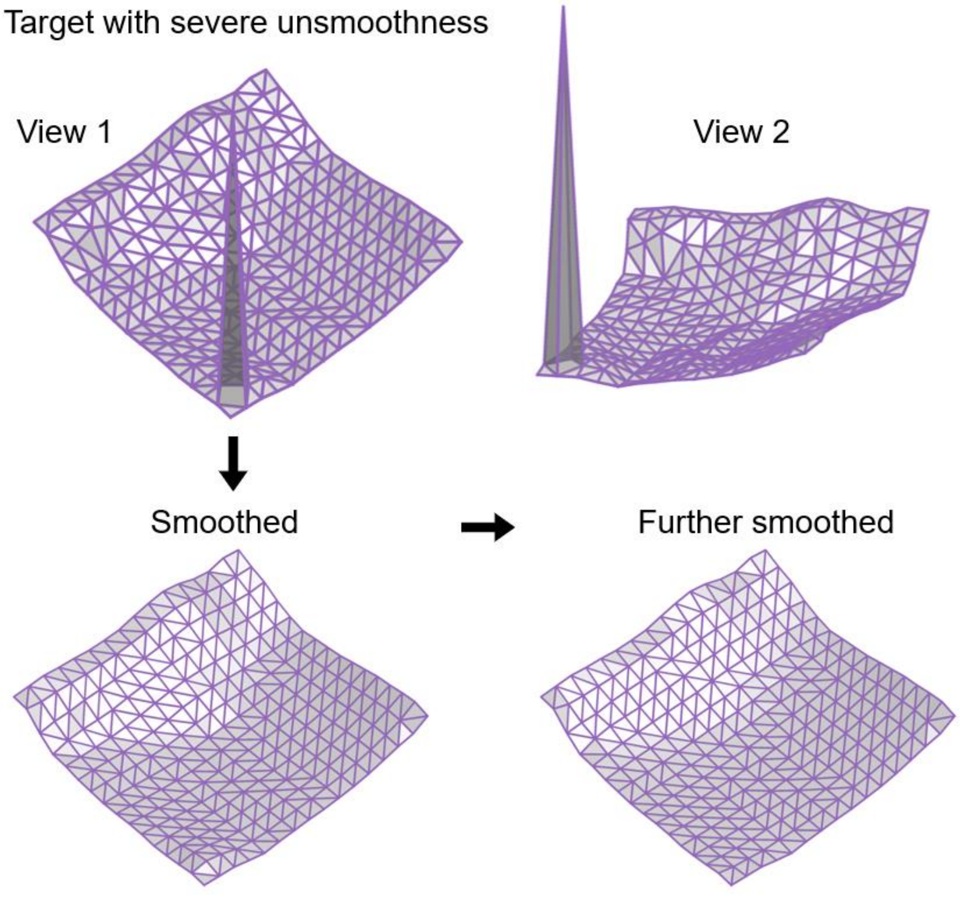

Supplementary Figure 13. The crumpled paper target shape with the severe unsmooth feature being intentionally introduced (top), the smoothed target shape with the unsmoothness being removed (bottom left), and the further smoothed target shape (bottom right).

Supplementary References

1. Kipf, T.N. and M. Welling, *Semi-supervised classification with graph convolutional networks.* arXiv preprint arXiv:1609.02907, 2016.
2. Li, G., et al. *Deepgcns: Can gcns go as deep as cnns?* in *Proceedings of the IEEE/CVF international conference on computer vision*. 2019.
3. Chen, M., et al. *Simple and deep graph convolutional networks*. in *International conference on machine learning*. 2020. PMLR.
4. Yang, Z., C.-H. Yu, and M.J. Buehler, *Deep learning model to predict complex stress and strain fields in hierarchical composites.* Science Advances, 2021. **7**(15): p. eabd7416.
5. Kumar, S., et al., *Inverse-designed spinodoid metamaterials.* npj Computational Materials, 2020. **6**(1): p. 73.
