# Supplementary material for: Machine learning-enabled forward prediction and inverse design of 4D-printed active plates
Source: Nat Commun. 2024 Jun 29;15:5509. doi: 10.1038/s41467-024-49775-z (PMC11217466; doi:10.1038/s41467-024-49775-z)
Supplement: Supplementary file 4 — Description of Additional Supplementary Files [file 41467_2024_49775_MOESM4_ESM.pdf]

Description of Additional Supplementary Files

File Name: **Supplementary Movie 1**

Description: 4D-printed, actuated shapes of given and optimized designs for the finite element-derived target shape 1 (row 1 of **Figure 5**).

File Name: **Supplementary Movie 2**

Description: 4D-printed, actuated shapes of given and optimized designs for the finite element-derived target shape 2 (row 2 of **Figure 5**).

File Name: **Supplementary Movie 3**

Description: 4D-printed, actuated shapes of given and optimized designs for the finite element-derived target shape 3 (row 3 of **Figure 5**).

File Name: **Supplementary Movie 4**

Description: 4D-printed, actuated shape of the optimized design versus 3D-printed target for the twisted parabolic target shape (bottom row of **Figure 6**).

File Name: **Supplementary Movie 5**

Description: 4D printing for the design validation on a smaller length scale in the twisted parabolic case (bottom row of **Figure 6**). As-printed shape, shape-morphing process and actuated shape are shown.

File Name: **Supplementary Movie 6**

Description: 4D printing for the design validation with two alternative material systems in the twisted parabolic case (bottom row of **Figure 6**). As-printed shape, shape-morphing

process and actuated shape are shown.

File Name: **Supplementary Movie 7**

Description: A complete design-fabrication process for 4D printing in the crumpled paper case (**Figure 7c**), including the paper crumpling, target identification, inverse design, 3D printing, shape morphing, and final actuated shape.

File Name: **Supplementary Movie 8**

Description: 4D-printed, actuated patch shape of the optimized design versus 3D-printed target for the surgical mask (bottom row of **Figure 7d**).
